# Associations between Absolute and Relative Lower Body Strength to Measures of Power and Change of Direction Speed in Division II Female Volleyball Players

**DOI:** 10.3390/sports7070160

**Published:** 2019-07-01

**Authors:** Whitney Tramel, Robert G. Lockie, Keston G. Lindsay, J. Jay Dawes

**Affiliations:** 1 Human Physiology and Nutrition, University of Colorado Colorado Springs, Colorado Springs, CO 80918, USA; 2 Department of Kinesiology, California State University, Fullerton, Fullerton, CA 92831, USA; 3 School of Kinesiology, Applied Health, and Recreation, Oklahoma State University, Stillwater, OK 74044, USA

**Keywords:** agility, acceleration, women’s sports, pre-season testing, strength training and conditioning

## Abstract

Volleyball is a sport comprised of multiple explosive efforts and multidirectional change of direction speed (CODS) actions. Since strength underpins both of these abilities, it is important to explore the relationship between these variables in order to develop strength and conditioning programs to optimize performance. The purpose of this study is to determine if a relationship exists between absolute and relative strength and measures of power and CODS in collegiate volleyball players. Archived testing data from ten (n = 10, age: 19.1 ± 1.2 yrs, Ht: 173.1 ± 6.64 cm, Wt: 67 ± 7.04 kg) female DII collegiate volleyball players were analyzed. These data included: block vertical jump (Block VJ), approach vertical jump (Approach VJ), a repeat jump test (i.e., four consecutive VJs), modified T-test, 5-0-5 agility test, a single leg triple hop test, and a 1-3RM deadlift. Significant large correlations were observed between relative strength and the repeat jump test, modified T-test, and 5-0-5 agility test. Significant correlations were also observed between absolute strength and the modified T-test. These results indicate that strength and conditioning professionals should emphasize the development of both absolute and relative lower-body strength to improve measures of power and agility in collegiate volleyball players.

## 1. Introduction

Volleyball is a sport comprised of multiple explosive efforts and multidirectional court movements that occur repeatedly during competition [1]. In order to be successful, players are frequently required to perform maximal effort jumps when blocking, spiking, and performing a jump serve [2]. Furthermore, they must be able to quickly position themselves on the court when responding to, or setting up, for an attack [1]. Thus, change of direction speed and agility are imperative for the volleyball athlete [3]. Indeed, because strength has been reported to underpin power and speed [4], one may posit that possessing adequate lower-body strength is also an essential physical quality for volleyball players to optimize performance.

Significant relationships between lower-body strength and change of direction speed (CODS) have been observed among athletes in a variety of sports. In a study by Anderson et al. [5], significant correlations were found between absolute (ABS) and relative (REL) strength and CODS as measured by the 5-0-5 CODS test (ABS: right leg: *r* = −0.51, *p* < 0.05; left leg: *r* = −0.59, *p* < 0.05; REL: right leg: *r* = −0.58, *p* < 0.05; left leg: *r* = −0.67, *p* < 0.01) and the modified T-test (Mod T; ABS: *r* = −0.55, *p* < 0.05; REL: *r* = −0.75, *p* < 0.01) in a group of female collegiate soccer players. Additionally, Nimphius et al. [6] discovered significant relationships between REL and measures of linear speed and CODS (*r* = −0.73–0.85, *p* < 0.05) in female softball players. However, limited research exists on the relationship between lower-body strength and CODS among female collegiate volleyball players. With the consistent offensive and defensive maneuvers volleyball players perform in a game, CODS becomes an integral aspect of the game [7]. Based on these findings, it is important to determine whether similar relationships exist between selective measures of strength and CODS performance in female volleyball players. Understanding these relationships may assist in the development of training programs to increase on-court performance within this population. 

Lower-body strength may play a significant role in a volleyball player’s ability to perform maximal effort jumps in competition [1]. Previous studies performed in male athletes have discovered significant correlations between relative strength and countermovement jump (CMJ) height (*r* = 0.690, *p* < 0.05) [8], as well as standing and run-up vertical jump (VJ) performance (*r* = 0.55–0.82, *p* < 0.05) [9]. However, no significant correlations were observed when ABS was compared to jump height. Nonetheless, multiple studies have concluded that participation in a resistance training program resulted in increases in CMJ height. In a study by Marques et al. [10], the researchers found that involvement in a 12-week in-season resistance training program led to significant increases in dynamic strength, measured by a four-repetition maximum in the bench press and back squat, and countermovement jump (CMJ) performance in a group of ten senior female professional volleyball players. Furthermore, research conducted by Fry et al. [11] discovered that participation in a 12-week off-season training program, consisting of four resistance training sessions and two plyometric sessions per week, led to significant increases in lower-body strength in the back squat and hang power clean, vertical jump (VJ) height and running VJ height. These findings suggest improvements in lower-body strength may have a positive influence on the physical qualities associated with successful on-court performance. However, at this time limited research exists showing the relationships between block and approach vertical jump and lower-body strength in female collegiate volleyball players. Additionally, to the best of the investigator’s knowledge, previous research has not been performed on the four-jump test in Division II female volleyball athletes. 

While previous studies have revealed the positive effect of a strength and conditioning program on jump performance in volleyball players, there exists a significant gap in the literature as it relates to correlations between predictors of performance, specifically CODS, and lower-body strength in female volleyball athletes. Therefore, the purpose of this study was to determine if significant relationships existed between absolute and relative lower-body strength and selected measures of power and CODS among Division II female volleyball players. It was hypothesized that both ABS and REL strength would be significantly correlated to all measures of power and CODS. 

## 2. Materials and Methods

### 2.1. Participants

Archived pre-season testing data for ten (n = 10) Division II collegiate women’s volleyball players (age = 19.1 ± 1.2 years; height =173.1 ± 6.64 cm; body mass = 67 ± 7.04 kg) were used for this analysis. Permission to use archived testing data was obtained from the athletes at the beginning of the academic year via the athletic department at the university in which this investigation occurred. Due to the archival nature of these data, the study qualified for exempt review via the Institutional Review Board (IRB) for human subjects at the University of Colorado, Colorado Springs (Ethical code #16-021).

### 2.2. Protocol

The pre-season fitness testing took place over two days, with 48 h rest between testing sessions. Each session included a standardized dynamic warm-up consisting of calisthenics and light jogging. During the first testing session, the following tests were performed on a hardwood volleyball court: block vertical jump (BVJ), approach vertical jump (AVJ), repeated jump test (four consecutive jumps), the single-leg triple hop (SLTH), the 505 CODS test, and the modified T test. The second testing session consisted of the three-repetition maximum hex bar deadlift (3RM).

### 2.3. Body Mass

At the beginning of the first testing, session height (cm) and body mass (kg) were measured for each athlete while barefoot, using a doctor’s beam scale (Cardinal; Detecto Scale Co, Webb City, MO, USA). Body mass was used in this study to calculate the relative strength of each athlete.

### 2.4. Block and Approach Vertical Jump

The BVJ and AVJ are commonly used lower-body power test among volleyball players [9]. Both the BVJ and AVJ were collected using a Vertec Jump Training System (Vertec Scientific Ltd, Aldermaston, United Kingdom). Prior to testing, the athletes were familiarized with the Vertec and given both verbal instructions and visual demonstrations for both jumps and their standing reach height was measured. For the BVJ, athletes were instructed to stand beneath the Vertec and when ready, perform a countermovement jump with an arm swing, and attempt to jump as high as possible, hitting the fins of the Vertec with both hands. This movement replicates deflecting a ball at the net from an opponent’s attack hit. Each athlete was allowed three attempts to achieve their maximal vertical jump height. Upon completion of the BVJ, all subjects performed the AVJ. For the AVJ, subjects were allowed a three-step run-in and were instructed to hit the highest fin possible with their dominant hand using a preferred take off strategy. Similar to the BVJ, athletes were allowed three attempts to achieve their best possible jump. To determine vertical jump height for both tests, the athletes standing reach was subtracted from the greatest height achieved for each jump. Each jump occurred with approximately 30–60 s rest between trials, which provided players enough time to recover. Due to time constraints, athletes were limited to three attempts. The best of three attempts was recorded to the nearest half inch, then converted to centimeters for analysis.

### 2.5. Repeat Jump Test

To measure sustained power, a repeated jump test was performed on a switch mat (Just Jump, Pro Biotics Inc., Hunstville, AL, USA). Previous research has found the Just Jump Mat system to be a valid method (R = 0.906) for measuring vertical jump height [12]. The repeated jump test used in this investigation required athletes to perform four countermovement jumps in a row while keeping the hands on their hips. Athletes were instructed to take off and land from the same position in the center of the mat. If the athlete’s landing deviated more than 6 inches, they were instructed to repeat the jumps. The average vertical jump height for all four jumps were recorded to the nearest tenth of an inch, and was then converted to metric units (i.e, centimeters) for the final analysis. 

### 2.6. The 505-CODS Test

**Figure 1:**
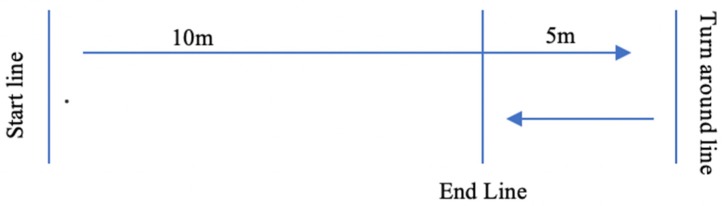
Set-up and execution of 505-CODS Test

The 505-CODS test (Figure 1) has been described in detail by Sheppard et al. [4]. Athletes were allowed approximately 2-minute rest between sets to minimize the impact of fatigue. Further, the order of the trials (i.e., left or right leg lead) were randomized to reduce any potential order effects utilizing by drawing numbers from a hat. A total of two trials were recorded per side with the two best scores from each side being used for this analysis.

### 2.7. Modified T-Test

**Figure 2:**
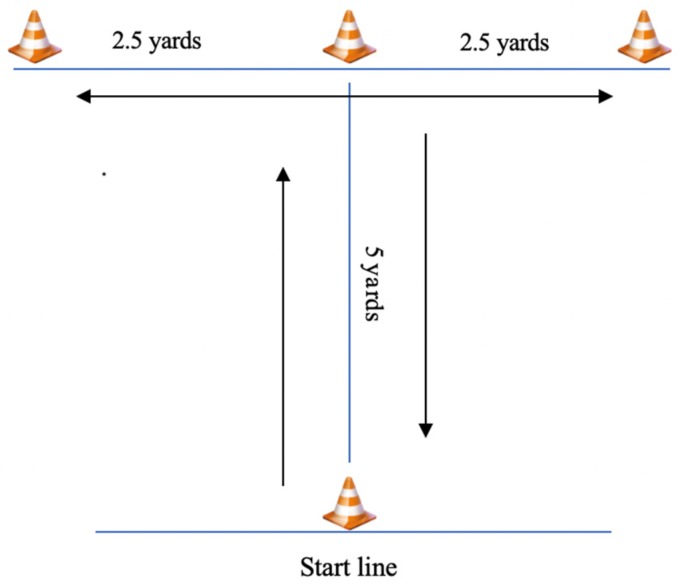
Set-up and execution of Modified T-Test.

This assessment was selected due to its similarity to the multiple changes of direction (i.e., linear acceleration, backpedaling and side shuffling) often seen during a rally. The protocol for this test (Figure 2) has been described in previous investigations [5] The best of two trials was recorded and rounded to the nearest tenth of a second.

### 2.8. Single Leg Triple Hop Fore Distance

The Single Leg Triple Hop for Distance (SLTH) test has been described in detail by Williams et al. [13]. Each athlete started by standing on their designated test leg with their toe on the starting line. When ready, the athlete was instructed to perform three consecutive maximal hops forward with the designated leg. The distance measured includes the distance hopped from the start line to the point where the subject’s heel lands on completion of the last hop. The purpose of this test is to measure unilateral power off both the right and left leg. 

### 2.9. Hex Bar Deadlift

Lower-body strength was measured using a 1-3RM hex bar deadlift test. Each athlete performed several warm up sets with progressively heavier loads. The specific warmup protocol for this test has been detailed in previous research [5]. Relative strength (REL) was calculated for each athlete by dividing their estimated 1 RM total and their body mass.

### 2.10. Statistical Analysis

Data were analyzed using a free open source statistical software package (JASP, Version 0.9.2, Amsterdam, NL USA). Descriptive statistics (mean ± SD) were calculated for each of the measured variables. A Pearson’s correlation coefficient was then used to relate the ABS and REL lower-body strength measures to scores on the selected performance tests. Statistical analysis was set at the a priori p ≤ 0.05 level. The strength of each correlation value was based on the criteria recommended by Hopkins [14]: 0 to 0.30, or 0 to −0.30 was considered low; 0.31 to 0.49, or −0.31 to −0.49 moderate; 0.50 to 0.69, or −0.50 to −0.69 large; 0.70 to 0.89, or −0.70 to −0.89 very large; and 0.90 to 1.0, or −0.90 to 1.0 a near perfect.

## 3. Results

Descriptive data for the subjects are shown in Table 1. The correlation data are displayed in Table 2. Significant large correlations were found between ABS and the modified T-test, and between REL and the Repeat Jump Test, Modified T Test, 505 R, 505 L, and SLTH L. There were no statistically significant correlations found between absolute strength and BVJ, AVJ, Repeat Jump Test, 505 R, 505 L, SLTH R, and SLTH L. No statistically significant correlations between relative strength and BVJ, AVJ, and SLTH R were discovered. 

## 4. Discussion

The purpose of this study was to determine if significant relationships existed between ABS and REL lower-body strength and measures of power and CODS among Division II female volleyball players. Strong significant correlations were observed between ABS and REL and CODS in the Modified T-test. Additionally, strong significant correlations between REL and repeat jump ability and the 505 CODS test were also observed. Neither the BVJ nor AVJ tests were correlated with strength. These results suggest that increasing absolute and relative lower-body strength may help improve CODS and repeat jump ability in collegiate volleyball players. 

The ability to change direction rapidly and efficiently is essential for success in the sport of volleyball [1]. Previous authors have noted the relationships between lower-body strength and CODS performance [4,15]. In the present study, significant correlations were found between both absolute and relative strength and CODS as measured by the Modified T-test. These results are similar to the findings of Andersen et al. [5] who discovered significant moderate-strong relationships between absolute and relative lower-body strength (3RM back squat) and CODS as measured by the Modified T-test and the 505 test. Keiner et al [16] also found significant moderate to strong relationships (*r* = −0.388 to −0.697, *p* < 0.05) between REL in the front and back squat and CODS among elite youth soccer players. Similarly, Peterson et al. [17] found significant associations between the T-test and REL as measured by the 1RM back squat. The results of this study, in conjunction with previous findings, suggest that the development of lower-body strength may improve CODS in collegiate volleyball players. 

Lower-body power is a highly desirable trait in the sport of volleyball [18,19]. Previous studies have reported significant moderate relationships between lower-body strength and jump height in elite volleyball players [2]. Young et al. [9] found significant correlations between standing and run-up vertical jump height when maximum strength was expressed relative to body mass, but not in absolute terms. However, in the current investigation, no significant correlations were discovered between lower-body strength and jump height in the BVJ or AVJ. This contrasts the findings of Kraska et al. [20] where researchers evaluated a trained group of Division I athletes from a variety of sporting backgrounds and discovered that maximal strength correlated with greater explosiveness and jump heights. Athletes in the current investigation may have utilized a force-dominant jumping strategy. In other words, these athletes may have relied more on strength to perform jumps, in lieu of effective utilization of the stretch-shortening cycle (SSC) [21]. Young et al. [9] found that reactive strength from a drop jump correlated more strongly with a run-up VJ than a standing VJ. A drop jump measures an athlete’s ability to effectively utilize the SSC producing greater power in a short amount of time (<0.25 s) [9]. Although a drop jump was not performed in the current investigation, this information can further support the athlete’s ineffective use of the SSC. 

Volleyball players must frequently perform repeated maximal or near maximal jumps, during a rally [1,3]. However, to the best of the investigator’s knowledge, repeat jump performance has not been thoroughly investigated in this population. While no significant relationships were discovered between lower-body strength and vertical jump height in this investigation, moderate correlations were observed between repeat jump performance and measures of REL. When performing repeated jumps, athletes need to be able to quickly utilize the stretch-shortening cycle to rapidly create force and limit ground contact time, resulting in greater vertical acceleration [2]. Therefore, in order to optimize repeat jump performance, it is recommended that athletes engage in training aimed at developing relative strength. With a higher strength:body mass ratio, athletes may be able to redirect their weight more quickly and efficiently. This would yield greater force production and shorter ground contact times resulting in the ability to jump higher repeatedly. 

The current investigation contributes to the overall body of knowledge relating lower-body-strength to CODS and repeat jump performance among collegiate female volleyball players. However, this study is not without limitations. The present study used archived data from one team at one point in time. Furthermore, data related to the individual athletes’ training age and playing experience were unavailable, which may be a useful variable to analyze in future research. Another limitation was the small sample size utilized for this analysis. Future research should examine data collected over different seasons and time points (i.e., preseason, in-season, offseason), and investigate the underlying mechanisms that may further explain the observed relationships. Additionally, future investigations should attempt to identify how these specific attributes contribute to an athlete’s success on the court. 

## 5. Conclusions

The results of the present study indicate that the development of lower-body strength, especially relative strength, may improve CODS and repeat jump performance in female collegiate volleyball players. Strength and conditioning coaches can utilize this information when developing training programs by incorporating training methods that will increase both absolute and relative strength. While relative strength may appear to have a greater impact on performance, the development of absolute strength can lead to further increases in relative strength, so long as there is no significant increase in an athlete’s body mass. It is important to recognize that although absolute strength did not show to have a strong correlation to jump performance, it may have a great influence on characteristics considered to be highly influential to jump performance, such as the ability to rapidly produce force, making the athlete more explosive. Further, it is recommended that athletes use other forms of resistance training, such as plyometrics, that emphasize the development of reactive strength and eccentric loading via short ground contact times. 

## Figures and Tables

**Table 1:** Descriptive statistics of participants and overall team averages for each performance variable (n = 10).

Variable	Team Average ± SD
Age (yrs)	19.10 ± 1.197
Height (cm)	173.1 ± 6.641
Body mass (kg)	67 ± 7.035
DL Est 1 RM (kg)	102.3 ± 20.07
Relative DL (DL kg/ BM kg)	1.54 ± 0.923
BVJ (cm)	51.69 ± 5.52
AVJ (cm)	60.33 ± 6.14
Repeat Jump Test	18.16 ± 2.064
Modified T-test (s)	6.76 ± 0.273
5-0-5 R (s)	5.245 ± 0.223
5-0-5 L (s)	5.21 ± 0.211
SLTH R (cm)	204.7 ± 14.83
SLTH L (cm)	205.3 ± 1606

**Table 2:** Relationship between lower-body strength and selected measures of performance.

Variable	ABS	REL
BVJ (cm)	−0.032	−0.118
AVJ (cm)	−0.195	−0.17
Repeat Jump Test	0.361	0.673 *
Modified T-test	−0.728 *	−0.84 **
5-0-5 R	−0.566	−0.689 *
5-0-5 L	−0.606	−0.743 *
SLTH R (cm)	0.185	0.474
SLTH L (cm)	0.402	0.668*

* *p* < 0.05, ** *p* < 0.01.
